# Protective effect of sildenafil on the genotoxicity and cytotoxicity in apolipoprotein E-deficient mice bone marrow cells

**DOI:** 10.1186/s12944-016-0268-6

**Published:** 2016-05-27

**Authors:** Franciane P. Bernardes, Alan T. Batista, Marcella L. Porto, Elisardo C. Vasquez, Bianca P. Campagnaro, Silvana S. Meyrelles

**Affiliations:** Laboratory of Translational Physiology, Health Sciences Center, Federal University of Espirito Santo, Vitoria, Brazil; Federal Institute of Education, Science and Technology (IFES), Vila Velha, ES Brazil; Pharmaceutical Sciences Graduate Program, Vila Velha University (UVV), Vila Velha, ES Brazil

**Keywords:** Atherosclerosis, ROS, Micronucleus, DNA, Genotoxicity

## Abstract

**Background:**

The pharmacological inhibitor of phosphodiesterase 5 (PDE5), sildenafil, is a promising candidate for antioxidant therapy that can result in cardiovascular protection. In addition to its known effects on the cardiovascular system, hypercholesterolemia leads to increased oxidative stress and DNA damage in the bone marrow, which is a non-classical target organ of atherosclerosis. In the present study, we evaluate oxidative stress and assess the effect of genomic instability on cell cycle kinetics in atherosclerotic animals and determine if sildenafil reverses these detrimental effects in bone marrow cells.

**Methods:**

Experiments were performed in male wild-type (WT) and apolipoprotein E knockout mice (apoE^−/−^) (9 weeks of age). apoE^−/−^ mice were randomly distributed into the following 2 groups: sildenafil-treated (40 mg/kg/day for 3 weeks, *n* = 8) and vehicle-treated (*n* = 8), by oral gavage. After treatment, bone marrow cells were isolated to assess the production of superoxide anions and hydrogen peroxide, determine cell cycle kinetics and evaluate the presence of micronucleated cells.

**Results:**

Sildenafil treatment reduced the cytoplasmic levels of superoxide anion (~95 % decrease, *p* < 0.05) and decreased hydrogen peroxide (~30 % decrease, *p* < 0.05). Moreover, we observed protective effects on the DNA of bone marrow cells, including normal cell cycling, decreased DNA fragmentation and a diminished frequency of micronucleated cells.

**Conclusion:**

Our data reveal that the excessive production of ROS in atherosclerotic mice overcome the DNA repair pathways in bone marrow cells. The novelty of the present study is that the administration of sildenafil reduced ROS to baseline levels and, consequently, reverted the DNA damage and its outcomes in bone marrow cells.

## Background

Hypercholesterolemia remains one of the most important risk factors for cardiovascular morbidity and mortality [1–4]. High plasma cholesterol levels trigger a cascade of events, including inflammation [5], oxidative stress [6] and cell damage [7, 8], particularly in the cardiovascular system [3]. Additionally, we have previously shown that hypercholesterolemia increases oxidative stress in different cell types [6, 7, 9, 10], including bone marrow mononuclear cells [8].

To understand the mechanisms underlying atherosclerosis development and progression and how it impacts cell function, we use apolipoprotein E deficient (apoE^−/−^) mice [1–3, 11], which show high levels of plasma cholesterol and develop atherosclerotic lesions that resemble human disease. Emerging evidence suggests the beneficial effects of phosphodiesterase 5 (PDE5) inhibition with sildenafil on cardiovascular diseases and other target organs [6, 7, 10, 12–14]. However, the effects of sildenafil on oxidative DNA damage in bone marrow cells from apoE^−/−^ mice have yet to be characterized.

Our study is the first work attempting to characterize the anti-oxidant effect of sildenafil on bone marrow cells as a means of protecting the main source of adult stem cells from damaging reactive oxygen species (ROS). This study has important implications for cardiovascular diseases, including atherosclerosis, a known risk factor for the development of cardiac, renal and vascular morbidities [6, 10].

In the present work, we assess oxidative stress and the link between genomic instability and cell cycle kinetics in atherosclerotic animals. Additionally, we evaluate the ability for sildenafil to prevent the damage incurred by ROS in bone marrow cells. To our knowledge, this is the first report demonstrating that chronic sildenafil administration prevents oxidative stress, genomic instability and cell cycle arrest in bone marrow cells in atherosclerotic mice.

## Methods

### Animals

The experiments were performed in 9-week-old male wild-type C57BL/6 (WT, *n* = 8) and apolipoprotein E-deficient (apoE^−/−^) mice. The animals were bred and maintained in the animal care facility at the Laboratory of Translational Physiology at the Federal University of Espirito Santo, Brazil. Mice were housed in individual plastic cages with a controlled temperature (22–23 °C) and humidity (60 %) and were exposed to a 12:12-h light-dark cycle. All mice were fed a standard chow diet and had access to water *ad libitum*. apoE^−/−^ mice were distributed into the following 2 groups: (*n* = 8 per group) sildenafil (Viagra®, Pfizer Laboratory, São Paulo, Brazil)-treated by oral gavage, 40 mg/kg/day for 3 weeks (Sil) and vehicle (saline)-treated. All experimental procedures were performed in accordance with the guidelines for the care and handling of laboratory animals as recommended by the National Institutes of Health (NIH), and the study protocols were previously approved by the Institutional Animal Care Committee (CEUA-UFES, Protocol # 008/2015).

### Analysis of plasma cholesterol and triglycerides

The total serum cholesterol and triglycerides from blood samples were determined using commercial colorimetric assay kits (Bioclin, Belo Horizonte, Brazil).

### Isolation of bone marrow cells

Bone marrow cells were obtained from the femurs and tibias of mice euthanized with a sodium thiopental overdose (100 mg/kg, i.p.). After cleaning all soft tissue, epiphyses were removed to gain access to the marrow cavities. Whole bone marrow was flushed out with DMEM, and the resultant cell suspension was incubated twice with a lysing buffer for 5 min at 37 °C to remove erythrocytes. The cell suspension was subsequently centrifuged for 10 min at 1200 rpm. The cells were counted and assessed for viability in Neubauer chamber [15]. The samples were considered viable when ≥ 90 % were found to be alive.

### Measurement of intracellular ROS

ROS analysis was performed by flow cytometry using dihydroethidium (DHE) and dichlorofluorescein diacetate (DCF) to detect intracellular •O_2_^−^ and H_2_O_2_, respectively, as previously described [8,16]. Briefly, DHE (160 mM) and DCF-DA (20 mM) were added to a cell suspension of 10^6^ cells and incubated at 37 °C for 30 min in the dark. The cells were kept on ice until flow cytometric acquisition (10,000 events; FACSCanto II, Becton Dickinson, San Juan, CA). The data were analyzed using FACSDiva software (Becton Dickinson) and were expressed as median fluorescence intensity (MFI).

### Cell cycle analysis

Cell cycle distribution in bone marrow cells was determined by flow cytometry analysis using propidium iodide (PI), as previously described [15]. Briefly, bone marrow cells were fixed, for 2 h, in cold 70 % ethanol and then incubated, for 30 min, with staining solution (20 mg/mL RNAse A, 500 mg/mL PI, 1 % Triton X-100). For determination of cell cycle distribution, samples were processed in triplicate (10,000 events; FACSCanto II flow cytometer). The cell cycle profile was determined via data analysis performed with FACSDiva software. The data are expressed as the percentage of cells in each cell cycle phase (sub-G_0_, G_0_/G_1_, S and G_2_/M).

### Micronucleus analysis

To evaluate the genotoxic risk of atherosclerosis and the anti-genotoxic activity of sildenafil in mice, the micronucleus test was performed on bone marrow cells. The experiments were performed according to von Ledebur and Schmid [17]. Briefly, bone marrow cells were flushed out from the humerus with foetal bovine serum. After centrifuging (1000 rpm, 10 min), cells were resuspended and a drop was smeared on individual glass slides. The samples were then fixed in methanol for 24 h and subsequently stained with Leishman stain. Slides were analyzed under a light microscope (×1000) to detect micronuclei in young red blood cells from each experimental group. The number of micronucleated polychromatic erythrocytes (PCE) was determined using two slides per animal and 1000 PCE per slide. The genotoxicity and antigenotoxicity were determined by comparing the number of micronucleated PCE in each group. In addition, cytotoxicity was evaluated by the ratio of PCE to normochromatic erythrocytes (PCE/NCE) in the first 200 cells observed [18].

### Statistical analysis

All data are shown as the mean ± SEM. The normality of the variables was previously analyzed using the Kolmogorov-Smirnov test. Because the data exhibited a Gaussian distribution, the statistical analysis was performed using the one-way analysis of variance (ANOVA). When the ANOVA showed significant differences, the Tukey’s test was performed as a *post hoc* analysis. The differences between the means were considered significant at *p* < 0.05.

## Results

### Plasma cholesterol and triglycerides

Table 1 shows the average values of total plasma cholesterol and triglycerides in WT and apoE^−/−^ mice treated with sildenafil or vehicle. As expected, cholesterol and triglyceride levels in apoE^−/−^ mice were ~5- and ~3-fold higher, respectively, than those in WT mice (*p* < 0.001). Sildenafil treatment did not change the values of these parameters.

**Table 1:** Plasma cholesterol and triglycerides

Parameter	WT	apoE^−/−^	Sildenafil
Total cholesterol (mg/dL)	113 ± 7	542 ± 10*	554 ± 15*
Triglycerides (mg/dL)	66 ± 4	192 ± 14*	184 ± 16*

The values are the means ± SEM

*p < 0.05 vs. WT group (one-way ANOVA and Tukey’s test)

### *Bone marrow cell counting and* viability

**Fig. 1:**
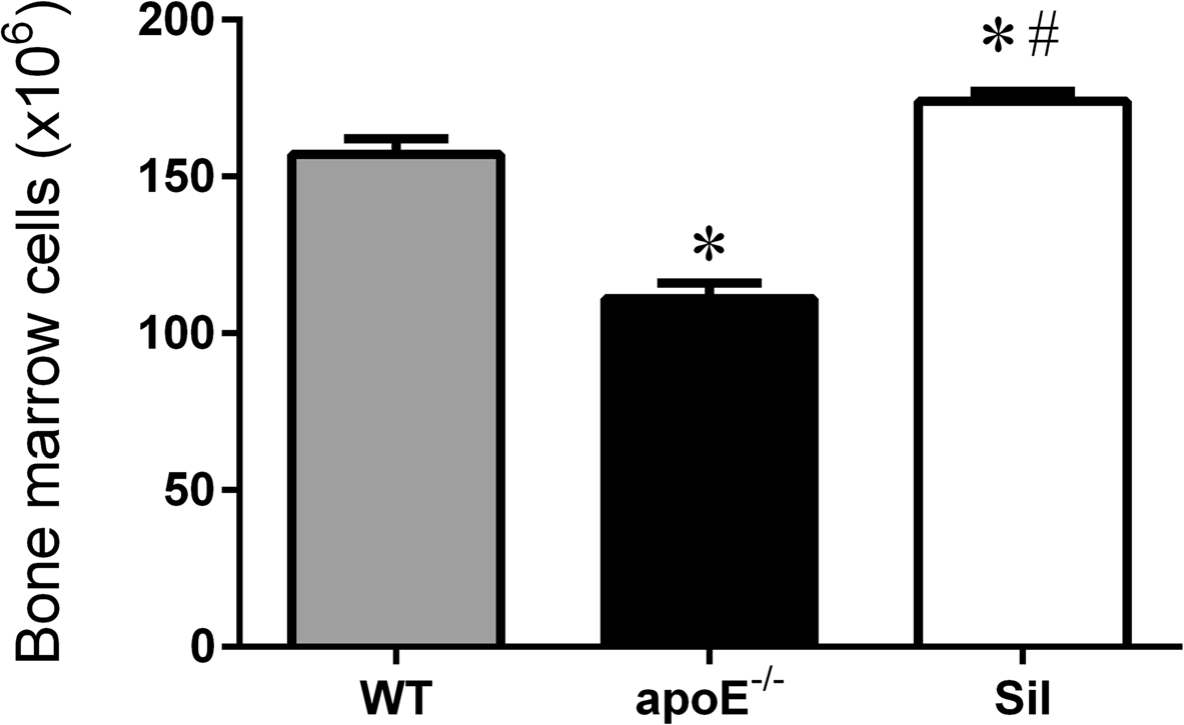
Number of bone marrow cells counted in WT, apoE^−/−^ and Sil mice using a Neubauer chamber. The values are the means ± SEM. *p < 0.05 vs. WT group; ^#^ *p* < 0.05 vs. apoE^−/−^ group (one-way ANOVA)

Figure 1 summarizes the average number of bone marrow cells counted in WT, apoE^−/−^ and Sil treated mice. apoE^−/−^ mice showed a decreased number of cells (110 ± 5, *p* < 0.01), in contrast with Sil mice, which exhibited an increased number of cells (173 ± 3 cells, *p* < 0.05) compared with WT mice (157 ± 5 cells). Cell viability (the total number of cells minus the number of dead cells) was ≥ 95 % in all three groups.

### ROS levels

**Fig. 2:**
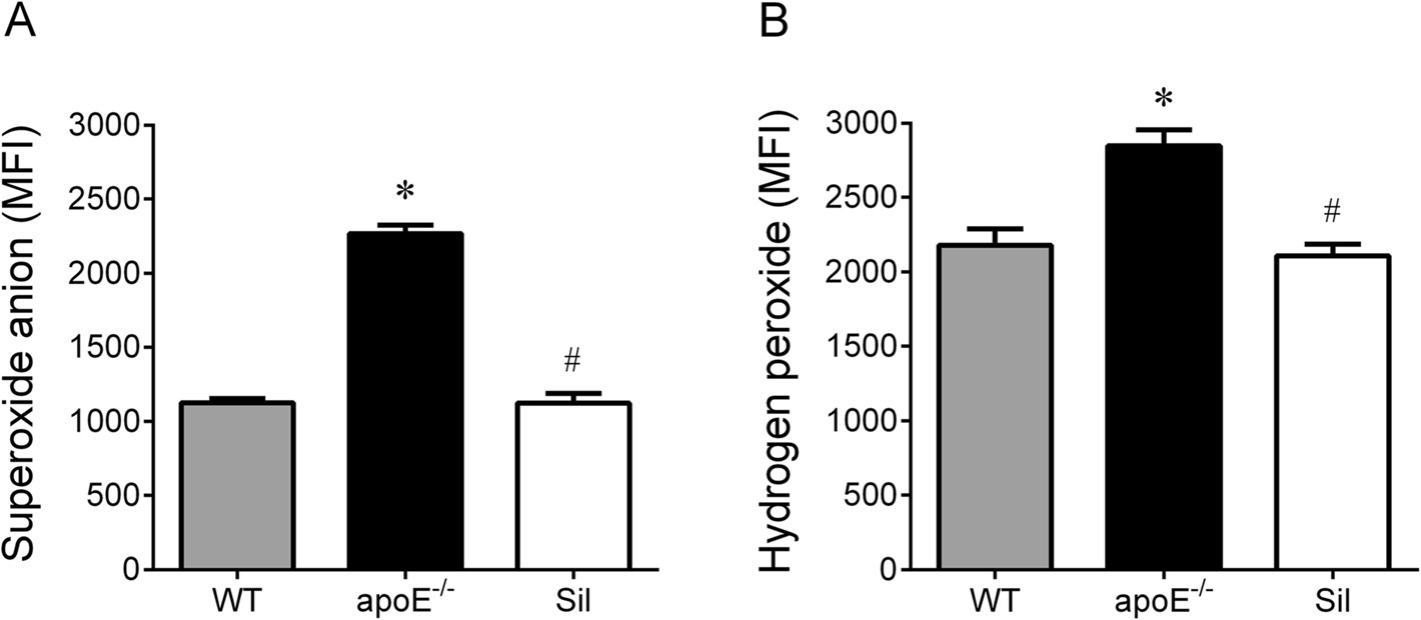
Levels of ROS in bone marrow cells using dihydroethidium (DHE) and dichlorofluorescein diacetate (DCF) to detect intracellular superoxide anion (**a**) and hydrogen peroxide (**b**), respectively, in WT, apoE^−/−^ and Sil mice. The values are the means ± SEM. *p < 0.05 vs. WT group; ^#^ *p* < 0.05 vs. apoE^−/−^ group (one-way ANOVA)

Figure 2 summarizes the MFI values of bone marrow cells •O_2_^−^ (Fig. 2a) and H_2_O_2_ (Fig. 2b) production, determined by flow cytometry, using DHE and DCF-DA fluorescent dyes, respectively. As expected, apoE^−/−^ mice exhibited a markedly augmented (+96 %, *p* < 0.01) production of •O_2_^−^ (2218 ± 360 MFI) compared with WT animals (1128 ± 28 MFI). These increased values were reduced to a level comparable to those found in WT controls upon treatment with sildenafil (1126 ± 190 MFI). The production of H_2_O_2_ was also significantly higher in the apoE^−/−^ group (30 %, *p* < 0.01) compared with WT animals (2181 ± 107 MFI) but was reduced upon sildenafil treatment to a level comparable with that found in WT mice (2107 ± 80 MFI).

### Evaluation of cell cycle distribution

**Fig. 3:**
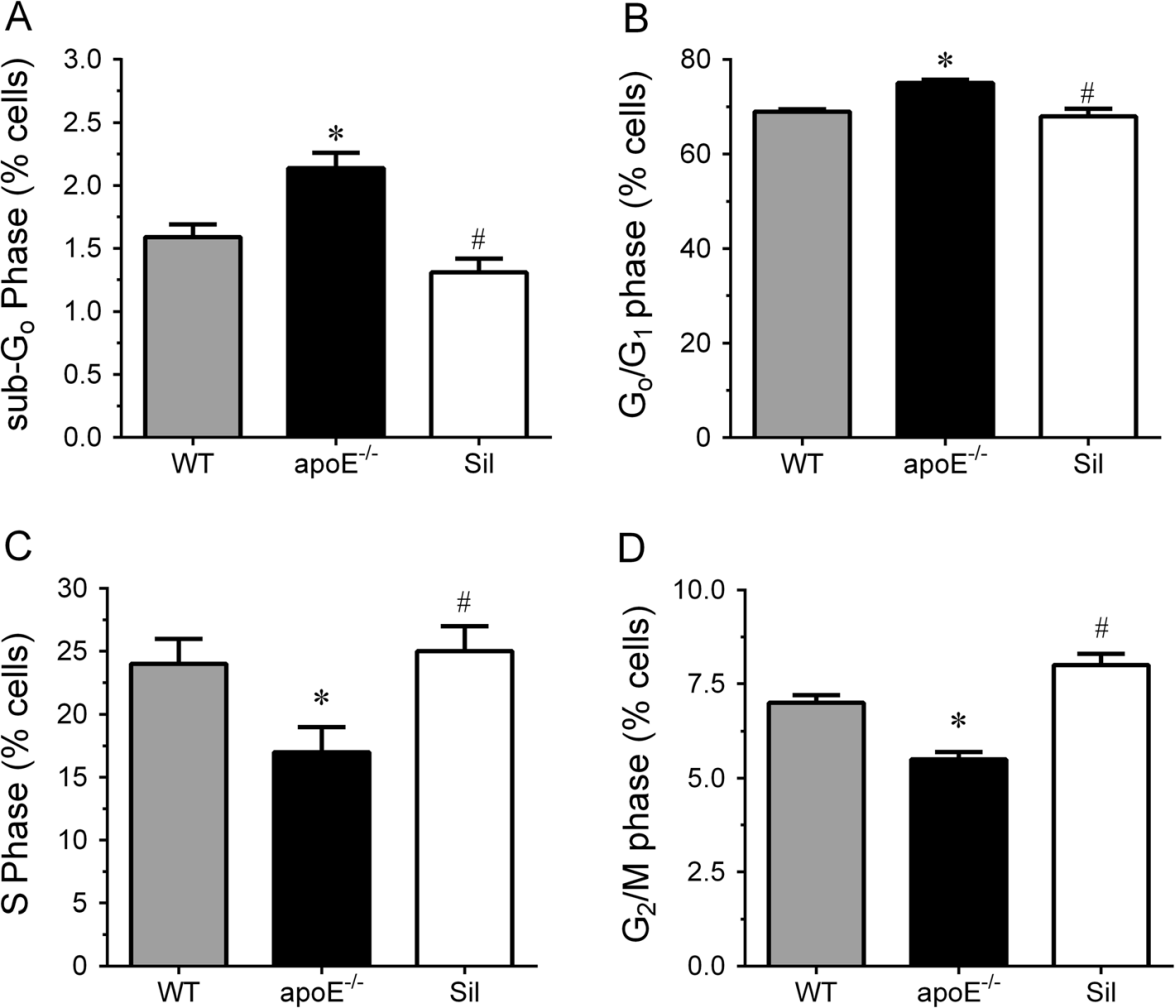
Effects of sildenafil treatment on cell cycle distribution in bone marrow cells from WT, apoE^−/−^ and Sil mice. The cell cycle distribution was monitored via PI staining and flow cytometric analysis. Statistical analysis of the percentage of cells in sub-G_0_-phase (**a**), G_0_/G_1_-phase (**b**), S-phase (**c**) and G_2_/M-phase (**d**). The values are the means ± SEM. *p < 0.05 vs. WT group; ^#^ *p* < 0.05 vs. apoE^−/−^ group (one-way ANOVA)

Considering that augmented ROS is known to cause damage to DNA, we evaluated cell cycle distribution by PI staining and flow cytometric analysis. We determined the percentage of cells with fragmented (sub-G0; × < 2n), interphasic (G0/G1; × = 2n), duplicating (S; 2n < × > 4n) and duplicated (G2/M; × = 4n) DNA. Figure 3 shows the percentage of cells in different phases of the cell cycle, including sub-G_0_ (Fig. 3a), G_0_/G_1_ (Fig. 3b), S (Fig. 3c) and G_2_/M (Fig. 3d). The percentage of bone marrow cells in sub-G_0_ and G_0_/G_1_ phases was significantly augmented in apoE^−/−^ mice compared with WT animals; these parameters returned to control group values in in apoE^−/−^ mice chronically administered with sildenafil (Sub-G_0_ – WT: 1.6 ± 0.10 % vs. apoE^−/−^: 2.2 ± 0.12 % vs. Sil: 1.3 ± 0.1 %, Fig. 3a; and G_0_/G_1_ – WT: 68 ± 0.5 % vs. apoE^−/−^: 75 ± 0.7 % vs. Sil: 67 ± 1.6 %, Fig. 3b). The percentage of bone marrow cells in S and G_2_/M phases was significantly diminished in apoE^−/−^ mice compared with WT animals; chronic administration with sildenafil returned these parameters to WT levels (S – WT:24 ± 2 % vs. apoE^−/−^: 17 ± 2 % vs. Sil: 25 ± 2 %, Fig. 3c; and G_2_/M – WT: 7 ± 0.2 % vs. apoE^−/−^: 5.5 ± 0.2 % vs. Sil: 8 ± 0.3 %, Fig. 3d).

### Micronucleus test

**Fig. 4:**
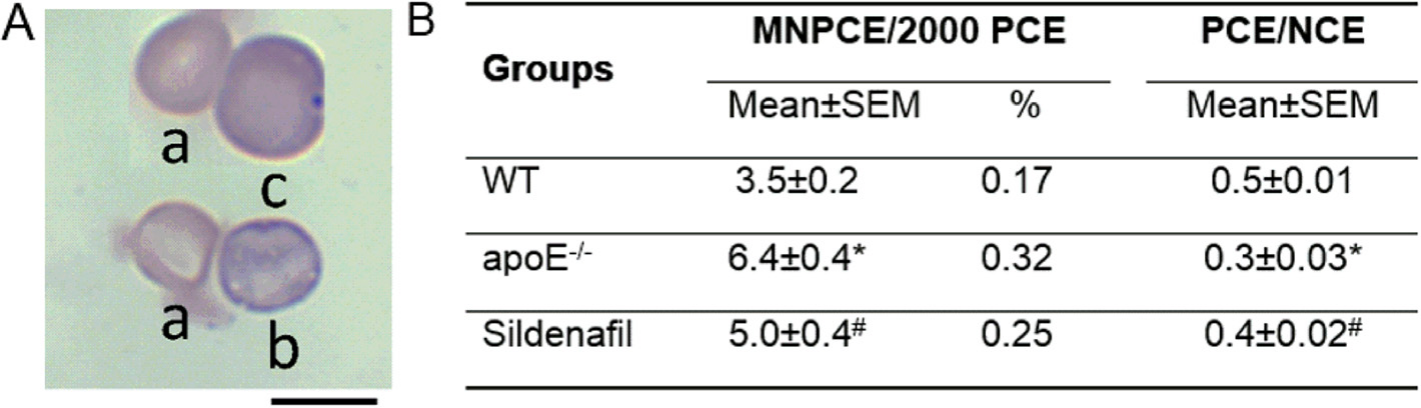
Evaluation of cytotoxicity and genotoxicity. **a** Typical photomicrographs of polychromatic (a; PCE) and normochromatic (b; NCE) erythrocytes, and micronucleated PCE (c; MNPCE) in bone marrow. **b** Table showing the effects of sildenafil treatment on the bone marrow of apoE^−/−^ mice based on MNPCE and PCE/NCE frequency. The values are the means ± SEM (*n* = 7 per group). * *p* < 0.05 vs. WT. ^**#**^ *p* < 0.05 vs. apoE^−/−^ (one-way ANOVA). Scale bar: 10 μm

In the present study, the micronucleus test was performed on bone marrow samples to investigate the protective effects of sildenafil on the DNA of apoE^−/−^ mice. Figure 4a shows typical images of PCE, NCE and micronucleated PCE (MNPCE) in bone marrow. A significant increase in micronuclei incidence was observed in apoE^−/−^ mice (6.4 ± 0.35 MNPCE) compared with WT mice (3.5 ± 0.27 MNPCE), and sildenafil treatment (5.0 ± 0.41 MNPCE) was able to reduce the frequency of micronucleated cells (Fig. 4b). In addition, the ratio of PCE/NCE reveals that atherosclerosis, per se, yields a cytotoxic effect compared with WT mice (WT: 0.52 ± 0.01 vs. apoE^−/−^: 0.28 ± 0.03). Additionally, sildenafil treatment protected bone marrow cells of apoE^−/−^ mice from the cytotoxic effect of hypercholesterolemia (0.42 ± 0.02).

## Discussion

This study shows, for the first time, that chronic inhibition of PDE5 with sildenafil restores cell cycle kinetics/progression and prevents genomic instability by decreasing ROS production in bone marrow cells of apoE^−/−^ mice. We have previously demonstrated the link between increased ROS production and DNA damage in bone marrow cells from apoE^−/−^ mice [8]. We have also demonstrated that sildenafil treatment was able to reduce DNA damage in blood, renal and liver cells [7, 12]. However, there is no evidence of sildenafil’s antioxidative effect on bone marrow cells and, consequently, on DNA damage. To explore this phenomenon, we evaluated the effects of sildenafil as an antioxidant, anti-genotoxic and, consequently, anti-mutagenic drug in the bone marrow cells of apoE^−/−^ mice.

Atherosclerotic mice showed higher plasma cholesterol and triglyceride levels than WT mice, corroborating recent findings [6, 7, 10]. The chronic administration of sildenafil did not change the plasma lipid profile in atherosclerotic animals, which is in agreement to previous results [6, 7, 10].

Oxidative stress has been implicated as a causative factor in cardiovascular diseases [18]. During atherosclerotic plaque development, cells accumulate high levels of ROS, which play an important role in disease initiation and progression [1–3]. Excess of ROS can trigger toxic effects by damaging biomolecules, which leads to cell death [7, 8, 12, 16]. In the present study, superoxide anion and hydrogen peroxide levels were augmented in bone marrow cells from apoE^−/−^ mice. These findings are in agreement with Tonini et al. [8], likely due to the hyperactivation of ROS-producing enzymes and the decreased activity of ROS-scavenger enzymes [19–23]. In addition, we demonstrated, for the first time, that chronic treatment with sildenafil was able to restore ROS production to a baseline level in bone marrow cells from apoE^−/−^ mice. It is known that sildenafil regulates NO/cGMP signaling, which has been implicated in the pathogenesis of atherosclerosis [6, 10]. Taken together, these data show that sildenafil reduces ROS production, without modifying the lipid profile of apoE^−/−^ mice.

ROS are well-known genotoxins that have been associated with DNA damage in cardiovascular diseases [8, 9, 12, 15]. It has been postulated that atherosclerosis leads to oxidative stress and could, indirectly, cause genomic instability, as excessive ROS lead to double-strand DNA breaks [7, 8]. The micronucleus test is a simple and quick technique commonly used for the detection of genotoxicity and cytotoxicity induced by chemical substances [24]. However, in the present study, we used the micronucleus test to investigate the mutagenic effects of hypercholesterolemia in bone marrow cells from atherosclerotic mice. The micronuclei are formed during anaphase due to a mismatch repair of double-strand breaks, which is the most common form of oxidative damage to DNA [25, 26]. We demonstrate a concurrent increase in ROS production and in MNPCE frequency in apoE^−/−^ mice compared with WT animals. Taken together, these data suggest that atherosclerosis is potentially genotoxic. On the other hand, the animals chronically treated with sildenafil exhibit MNPCE close to control levels, suggesting that sildenafil could be an anti-mutagenic drug due to its antioxidative activity. In addition, apoE^−/−^ mice showed higher cytotoxicity, evaluated by the ratio of PCE/NCE, compared with WT mice, suggesting that hypercholesterolemia presents cytotoxic action in bone marrow cells of atherosclerotic mice and sildenafil treatment completely abolishes this toxicity. The results of the micronucleus test suggest that chronic administration of sildenafil has anti-genotoxic, anti-cytotoxic and, consequently, anti-mutagenic effects due to its antioxidant activity in the bone marrow cells of atherosclerotic mice.

It is well known that elevated ROS levels alter cell cycle progression [27–32]. In the present study, we have found that oxidative stress change cell cycling in the bone marrow cells of atherosclerotic mice, as the bone marrow cells in apoE^−/−^ mice were less proliferative than in WT mice (more cells in G_0_/G_1_ phase, and less in S phase), suggesting that hypercholesterolemia induces a G_0_/G_1_ arrest in bone marrow cells. In addition, the percentage of bone marrow cells in S phase decreased in apoE^−/−^ mice and this was accompanied by a simultaneous increase in percentage of cells in G_0_/G_1_ phase, suggesting an inhibition in the progression of G1 to S phase.

The cells arrested in G_0_/G_1_ phase may die or they may be repaired and re-enter into the next phase of the cell cycle [33–36]. We observed more cells in the sub-G_0_ phase, due to DNA fragmentation, suggesting an increase in apoptosis in atherosclerotic mice [7, 8]. Interestingly, sildenafil treatment rescued cells from G_0_/G_1_ growth arrest and decreased DNA fragmentation [7], restoring the normal cell cycle distribution and protecting cells from undergoing apoptosis. Mammalian cells are subject to cycle checkpoints that allow DNA damage repair by slowing or arresting cell cycle progression, thereby preventing the transmission of damaged chromosomes [36–38]. We therefore suspect that the G_0_/G_1_ arrest observed in our apoE^−/−^ mice may be triggered by oxidative-DNA damage, which was prevented by sildenafil treatment.

To our knowledge, we are the first to use flow cytometry to show that chronic sildenafil treatment by oral gavage, in addition to its vasoactivity, indirectly prevents damage to bone marrow cells by reducing ROS production and genomic instability which, consequently, preserves cell cycle kinetics in different cell types from atherosclerotic and hypertensive mice [7, 10, 12–14]. Considering that the bone marrow is the primary stem cell source in adults and that autologous transplantation is the preferred therapy in clinical applications, our data provide insights into therapeutic potential of bone marrow stem cells from patients with cardiovascular disease.

## Conclusions

In conclusion, our data reveal that the excessive production of ROS in atherosclerotic mice overcome the DNA repair pathways in bone marrow cells. The novelty of the present study is that the administration of sildenafil reduced ROS to baseline levels and, consequently, reverted the DNA damage and its outcomes in bone marrow cells. Of note, there is a lot of efforts to discover a promising pharmacologic strategy to avoid tissue damage induced by oxidative stress. Therefore, further studies are needed to clear the mechanisms by which sildenafil acts as an anti-oxidant and anti-genotoxic drug.
